# MRI grading of spinal stenosis is not associated with the severity of low back pain in patients with lumbar spinal stenosis

**DOI:** 10.1186/s12891-022-05810-y

**Published:** 2022-09-12

**Authors:** Masakazu Minetama, Mamoru Kawakami, Masatoshi Teraguchi, Sachika Matsuo, Yoshio Enyo, Masafumi Nakagawa, Yoshio Yamamoto, Tomohiro Nakatani, Nana Sakon, Wakana Nagata, Yukihiro Nakagawa

**Affiliations:** 1 Spine Care Center, Wakayama Medical University Kihoku Hospital, 219 Myoji, Katsuragi-cho, Ito-gun, Wakayama, 649-7113 Japan; 2 Department of Orthopaedic Surgery, Saiseikai Wakayama Hospital, 45 Jyunibancho, Wakayama, 640-8158 Japan

**Keywords:** Lumbar spinal stenosis, Spondylolisthesis, Low back pain, Modic changes, Endplate defects, Disc degeneration, Facet joint osteoarthritis, Muscle mass, Spinal alignment, Magnetic resonance imaging

## Abstract

**Background:**

Although lumbar spinal stenosis (LSS) often coexists with other degenerative conditions, few studies have fully assessed possible contributing factors for low back pain (LBP) in patients with LSS. The purpose of this study was to identify factors associated with the severity of LBP in patients with LSS.

**Methods:**

The patients with neurogenic claudication caused by LSS, which was confirmed by magnetic resonance imaging (MRI) were included in this cross-sectional study. Data included ratings of LBP, buttock and leg pain, and numbness on a numerical rating scale (NRS), 36-item Short-Form Survey (SF-36) scores, muscle mass measured by bioelectrical impedance analysis, and radiographic measurements including lumbopelvic alignment and slippage. The severity of LSS, endplate defects, Modic endplate changes, intervertebral disc degeneration, and facet joint osteoarthritis were evaluated on MRI. Spearman correlation and multivariate linear regression analyses were used to examine the factors associated with the severity of LBP (NRS score).

**Results:**

A total of 293 patients (135 male and 158 female, average age 72.6 years) were analyzed. LBP was moderately correlated with buttock and leg pain, and buttock and leg numbness. Significant but weak correlations were observed between LBP and body mass index, appendicular and trunk muscle mass, all domains of SF-36, pelvic tilt, total number of endplate defects and Modic endplate changes, and summary score of disc degeneration grading, but not severity or number of spinal stenoses. In the multivariate regression analysis, age, female sex, trunk muscle mass, diabetes, NRS buttock and leg pain, NRS buttock and leg numbness, SF-36 vitality, pelvic tilt, and total number of endplate defects were associated with the severity of LBP.

**Conclusions:**

Trunk muscle mass, lumbopelvic alignment, and endplate defects, but not severity of stenosis are partly associated with severity of LBP, but buttock and leg pain and buttock and leg numbness have strongest relationships with LBP in patients with LSS.

## Background

Lumbar spinal stenosis (LSS) is a clinical syndrome of pain in the buttocks or lower extremities, with or without back pain, associated with diminished space available for the neural and vascular elements in the lumbar spine [1]. In fact, some patients with LSS do not complain of low back pain (LBP) [2]. To date, many studies have analyzed the relationship between abnormal magnetic resonance imaging (MRI) findings and the severity of symptoms, such as LBP, leg pain, and walking capacity in patients with LSS, but no or weak correlations between MRI findings and LSS symptoms have been reported in the previous study combined with systematic literature review and prospective cohort study [3].

Intervertebral disc, facet joint, vertebral body, nerves, and paraspinal musculature are commonly considered the source of LBP [4]. Recent attention has been focused on the endplate, which is more vascular and neural than the disc, as a potential source of LBP [5]. Previous studies showed that trunk muscle mass has also been associated with LBP in patients with spinal disorders or LSS [6, 7]. Furthermore, a systematic review and meta-analysis revealed that lumbopelvic alignment such as decreased lumbar lordosis (LL) has a strong relationship with LBP [8]. A large population study showed that LL decreases with age [9]. Another study reported that patients with LSS had less LL compared with age and sex-matched healthy subjects with LBP [10]. A correlation between decreased LL and reduced multifidus size in patients with LSS has been reported in a retrospective study [11].

Although LSS often coexists with other degenerative conditions [2], few studies have fully assessed these possible contributing factors, including endplate abnormalities, disc degeneration, facet joint osteoarthritis, lumbopelvic alignment, and trunk muscle mass for LBP in patients with LSS. Previous study investigated the factors associated with LBP, including these possible contributing factors, and found that the presence of endplate defects, but not spinal stenosis were associated with the presence of LBP in patients with LSS [12]. However, the factors associated with the severity of LBP have yet to be fully assessed. Given that multifactorial causes contribute to pathogenesis of LBP, it is crucial to identify to what extent each factor contributes to LBP. The purpose of this study was to clarify which factors are associated with the severity of LBP in patients with LSS.

## Methods

### Study design and population

This cross-sectional study was conducted at the Spine Care Center of Wakayama Medical University Kihoku Hospital from September 2017 to August 2021. The study received approval of the Institutional Review Board at Wakayama Medical University (No. 2378), and was performed in accordance with the Declaration of Helsinki. All participants provided written informed consent before enrollment.

The study protocol followed an earlier study that investigated the factors associated with the presence of LBP in patients with LSS [12]. The inclusion criteria were as follows: (1) > 50 years of age; (2) the presence of neurogenic intermittent claudication and pain and/or numbness in the lower extremities with or without LBP; (3) LSS confirmed by MRI; and (4) a referral to physical therapy. The presence of neurogenic intermittent claudication was confirmed from medical records of treating spine surgeon and physical therapist based on their history talking and physical assessments. Both patients who received outpatient physical therapy, and patients who scheduled for surgery and assessed preoperatively were included. The exclusion criteria were as follows: previous spine surgery; foraminal stenosis; spondylolysis; osteoarthrosis of the knee and/or hip; cognitive impairment; history of psychiatric illness; and prostheses or metal implant, or implants or devices that are contraindications for body composition analysis such as the presence of an electronic implant (e.g., heart pacemaker or brain stimulator). Consecutive patients meeting the inclusion and exclusion criteria were included in this study from September 2017 to August 2021.

### Measurements

The severity of LBP, buttock and leg pain, and buttock and leg numbness were measured using a numerical rating scale (NRS). The NRS questionnaire asked patients to rate their maximum LBP and, buttock and leg pain and numbness respectively from 0 to 10 during the past week. The location of LBP was defined between the lower edge of the ribs and the intercristal line (Jacoby’s line). The Medical Outcomes Study 36-Item Short-Form General Health Survey (SF-36) [13] was used to assess the patients’ health-related quality of life. Bioelectrical impedance analysis (BIA) was used to measure the appendicular and trunk skeletal muscle mass using an InBody S10 device (InBody Co. Ltd., Seoul, South Korea).

### Radiographic measurements

Orthopedic spine surgeons who were certified as specialists by the Japanese Orthopedic Association and Japanese Society for Spine Surgery and Related Spine Research evaluated the radiographic measurements and MRI findings. These surgeons were unaware of the study purposes and examined intra- and interrater reliability of the radiographic and MRI evaluations in 30 randomly selected patients.

Lumbopelvic alignment, including LL, pelvic incidence (PI), pelvic tilt (PT), and sacral slope were measured using standing lateral radiographs. The presence and percentage of slippage (% slip) were evaluated using lumbar flexion–extension radiographs of the patient in the standing position. The intra- and interclass correlation coefficient values for radiographic measurements were 0.98 and 0.96, respectively. The intra- and interobserver kappa values for the presence of slippage were 0.80 and 0.74, respectively.

### MRI evaluation

The lumbar spine was imaged using a 1.5-Tesla scanner (Signa HDxt 1.5 T, GE Healthcare, Chicago, IL). The axial images were acquired with a repetition time (TR)/echo time (TE) of 775 ms/14 ms and 3950 ms/110 ms for T1-weighted (T1W) and T2-weighted (T2W) images, respectively, field-of-view of 200 mm, matrix of 320 × 224, a slice thickness of 4 mm, and intersection gap of 1 mm. The sagittal images were acquired with a TR/TE of 670 ms/14.2 ms and 4300 ms/102 ms for T1W and T2W images, respectively, field-of-view of 300 mm, matrix of 384 × 256, a slice thickness of 4 mm, and intersection gap of 1 mm.

The grading of spinal stenosis was based on the rootlet/cerebrospinal fluid ratio as seen in axial T2W images according to the Schizas classification [14]. Grades A1–A4 indicate no or minor stenosis; grade B, moderate stenosis; grade C, severe stenosis, and; grade D, extreme stenosis. The intra- and interobserver kappa values were 0.77 and 0.68, respectively.

Endplate defects were categorized into three types (focal, corner, or erosive defects) according to the Feng classification on sagittal T2W images [15]. Ten endplates in the lumbar spine (L1 − S1) were examined for the presence or absence of any type of defect. The intra- and interobserver kappa values were 0.78 and 0.65, respectively.

Modic endplate changes were classified into three types (type 1, 2, or 3) [16]. The intra- and interobserver kappa values were 0.82 and 0.69, respectively.

Disc degeneration was examined using the Pfirrmann grading system (Grades I–V), with higher grades indicating more progressive degeneration [17]. The intra- and interobserver kappa values were 0.77 and 0.71, respectively.

Disc bulging and disc height narrowing were evaluated using a 0–3 rating scale, with 0 defined as normal and 1–3 representing progressive degrees of abnormality [18]. The intra- and interobserver kappa values were 0.71 and 0.57 for disc bulging and 0.80 and 0.70 for disc height narrowing, respectively.

Facet joint osteoarthritis was assessed on axial T1W images using the Fujiwara grading system (1–4) [19], where grade 1 indicates a normal joint and grade 4 indicates marked osteophytes. The intra- and interobserver kappa values were 0.76 and 0.65, respectively.

Severity of stenosis was determined at the maximum stenotic level (Grades A to D), and total numbers of spinal stenoses (more than grade B or C) were counted from L1/2 to L5/S. Each description of Modic endplate change, endplate defects, and disc degeneration (more than grade IV) [20], and facet joint osteoarthritis (more than grade 3) [21] were evaluated from L1/2 to L5/S. The scores for the grade of disc degeneration (1–5) [22], disc height, and disc bulging (0–3) [18] were summed from L1/2 to L5/S, because disc degeneration summary score has been reported to be associated with the presence of LBP [23].

### Statistical analysis

Spearman correlation analysis was used to identify relationships between the severity of LBP (measured by NRS score) and demographic data, muscle mass, and radiographic and MRI measurements. Correlation between a continuous variable and categorical variable such as sex was analyzed using a point-biserial correlation analysis. Stepwise linear regression analysis was used to examine the factors associated with the severity of LBP. The factors associated with buttock and leg pain, and buttock and leg numbness were also analyzed by correlation and regression analyses. Missing data were not imputed and assumed to be missing completely at random. All computations were performed using IBM SPSS Statistics for Windows (version 27.0; IBM Corp., Armonk, NY).

## Results

**Fig. 1:**
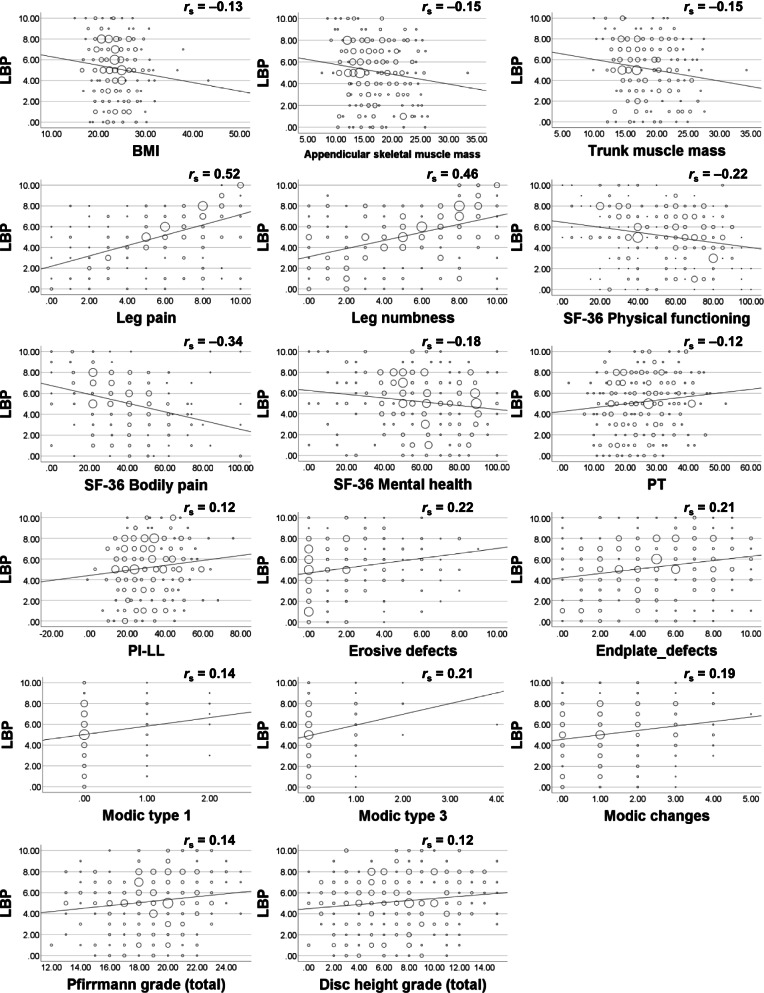
The factors correlated with LBP

Nine out of 302 enrolled patients had missing data in the SF-36 questionnaire, leaving a total of 293 patients (135 male and 158 female, average age 72.6 years) who were analyzed in this study. Forty-two patients were added from an earlier study [12]. Moderate correlations were observed between LBP and buttock and leg pain, and between LBP and buttock and leg numbness (Table 1, and Fig. 1). Significant but weak correlations were observed between LBP and body mass index, appendicular and trunk muscle mass, all domains of SF-36, PT, PI-LL, total number of endplate defects and Modic endplate changes, and summary score of disc grading and disc height grading, but not severity and the number of levels of spinal stenosis (Tables 1, 2, and Fig. 1). Stepwise linear regression analysis showed that age, female sex, trunk muscle mass, diabetes, NRS buttock and leg pain, NRS buttock and leg numbness, SF-36 vitality, PT, and total number of endplate defects were associated with the severity of LBP (Table 3).

**Table 1:** Correlations between demographics and NRS

	***N*** = 293	NRS LBP	NRS Buttock and leg pain	NRS Buttock and leg numbness
		***r***_**s**_	***r***_**s**_	***r***_**s**_
Age (years)	74 (68, 78)	−0.03	−0.04	0.02
Sex (male:female)	135:158	0.06	0.07	−0.11
Body mass index (kg/m^2^)	23.5 (21.2, 25.6)	−0.13*	− 0.03	− 0.14*
Appendicular skeletal muscle mass (kg)	16.0 (13.2, 20.1)	−0.15*	− 0.09	0.04
Trunk muscle mass (kg)	17.3 (15.0, 21.1)	−0.16*	−0.09	0.06
Duration of symptoms (months)	24 (11, 55)	0.07	−0.07	−0.01
Comorbidities (number, %)				
Hypertension	181 (61.8)	0.08	0.06	0.03
Diabetes	86 (29.4)	0.01	−0.10	−0.14*
Dyslipidemia	83 (28.3)	0.01	0.06	0.01
Heart disease	63 (21.5)	0.04	0.01	0.04
Pulmonary disease	24 (8.2)	0.01	0.01	0.03
Number of comorbidities	1 (1, 2)	0.07	0.05	0.02
NRS				
Back pain	5 (3, 7)		0.52*	0.46*
Buttock and leg pain	6 (4, 8)	0.52*		0.62*
Buttock and leg numbness	5 (3, 8)	0.46*	0.62*	
SF-36				
Physical functioning	55 (35, 70)	−0.22*	−0.27*	−0.22*
Bodily pain	32 (22, 51)	− 0.34*	− 0.48*	− 0.28*
Role physical	50 (25, 68.8)	− 0.18*	− 0.26*	− 0.13*
Role emotional	50 (33.3, 75)	−0.15*	−0.26*	− 0.10
Mental health	60 (47.5, 80)	−0.18*	− 0.26*	− 0.13*
Social functioning	62.5 (50, 87.5)	−0.15*	− 0.23*	− 0.10
Vitality	50 (31.3, 68.8)	−0.27*	−0.29*	− 0.15*
General health	50 (40, 60)	−0.26*	−0.20*	− 0.17*

Median (first quartile, third quartile). ***r***_s_, Spearman’s rank correlation coefficient, *P <  0.05

*NRS* numerical rating scale; *SF-36* Medical Outcomes Study 36-item Short-Form General Health Survey

**Table 2:** Correlations between radiological and MRI findings, and NRS

	***N*** = 293	NRS LBP	NRS Buttock and leg pain	NRS Buttock and leg numbness
		***r***_**s**_	***r***_**s**_	***r***_**s**_
LL (°)	21 (12, 31)	−0.05	0.04	−0.03
PI (°)	51 (44, 60)	0.09	0.10	< 0.01
PT (°)	25 (18, 32)	0.12*	0.04	−0.06
SS (°)	27 (22, 32)	−0.02	0.04	< 0.01
PI-LL (°)	30 (21, 41)	0.12*	0.02	0.02
Presence of slippage *n* (%)	126 (43.0)	−0.05	0.03	− 0.04
% Slip (%)	0 (0, 14.8)	−0.06	0.05	−0.04
Severity of stenosis (maximum stenotic level), (Grade A:B:C:D)	44:81:165:3	0.04	0.14*	0.19*
Total number of stenoses (more than grade B), *n* segments	2 (1, 3)	−0.02	0.05	0.11
Total number of stenoses (more than grade C), *n* segments	1 (0, 1)	0.01	0.07	0.13*
Total number of focal defects, *n* endplates (from L1 to S1, per patient)	1 (0, 3)	0.06	−0.02	< 0.01
Total number of corner defects, *n* endplates (from L1 to S1, per patient)	0 (0, 2)	0.06	0.02	0.04
Total number of erosive defects, *n* endplates (from L1 to S1, per patient)	1 (0, 3)	0.22*	0.11	0.16*
Total number of endplate defects, *n* endplates (from L1 to S1, per patient)	5 (3, 7)	0.21*	0.05	0.15*
Total number of Modic type 1, *n* segments (from L1/2 to L5/S, per patient)	0 (0, 0)	0.14*	0.07	0.19*
Total number of Modic type 2, *n* segments (from L1/2 to L5/S, per patient)	1 (0, 2)	0.05	0.03	−0.01
Total number of Modic type 3, *n* segments (from L1/2 to L5/S, per patient)	0 (0, 0)	0.21*	0.10	0.12*
Total number of Modic endplate changes, *n* segments (from L1/2 to L5/S, per patient)	1 (0, 2)	0.19*	0.10	0.12*
Summary score of disc degeneration grading	19 (17, 21)	0.14*	0.04	0.11
Total number of disc degeneration (more than grade IV), *n* segments	4 (2, 5)	0.09	0.04	0.09
Summary score of disc bulging grading	7 (5, 8)	0.07	0.11	0.19*
Summary score of disc height grading	7 (4, 10)	0.12*	−0.03	0.07
Total number of facet joint osteoarthritis (more than grade 3), *n* segments	3 (2, 4)	< 0.01	0.05	0.01

Median (first quartile, third quartile). ***r***_**s**_, Spearman’s rank correlation coefficient, *P <  0.05

*LL* lumbar lordosis; *PI* pelvic incidence; *PT* pelvic tilt; *SS* sacral slope

**Table 3:** Stepwise linear regression analysis to identify factors associated with severity of LBP

	Variable	*B*^a^	SE^b^	*β*^c^	*P*	Adjusted *R*^2^
Final	(Constant)	10.332	2.410			0.370
	Age	− 0.050	0.017	− 0.160	0.003	
	Female	−0.997	0.421	−0.192	0.018	
	Trunk muscle mass	−0.217	0.055	−0.335	< 0.001	
	Diabetes	0.556	0.271	0.098	0.041	
	NRS Buttock and leg pain	0.286	0.062	0.289	< 0.001	
	NRS Buttock and leg numbness	0.211	0.054	0.242	< 0.001	
	SF-36 Vitality	−0.014	0.006	−0.119	0.018	
	PT	0.030	0.014	0.105	0.030	
	Total number of endplate defects	0.191	0.047	0.197	< 0.001	

The independent variables entered into the model were age, sex, body mass index, presence and number of comorbidities, trunk muscle mass, appendicular skeletal muscle mass, NRS, SF-36 score, and radiological and MRI findings

^a^Unstandardized coefficient

^b^standard error

^c^standardized coefficients

Strong correlation was observed between buttock and leg pain and buttock and leg numbness (Table 1). Buttock and leg pain and buttock and leg numbness were weakly correlated with severity of spinal stenosis (Table 2). Buttock and leg numbness was also weakly correlated with number of spinal stenoses, total number of endplate defects and Modic endplate changes, and summary score of disc-bulging grading (Table 2). In the stepwise linear regression analyses, LBP, buttock and leg numbness, SF-36 bodily pain, and %Slip were associated with the severity of buttock and leg pain (Table 4). Age, body mass index, trunk muscle mass, LBP, buttock and leg pain, total number of Modic type 1 endplate changes, and severity of spinal stenosis were associated with the severity of buttock and leg numbness (Table 5).

**Table 4:** Stepwise linear regression analysis to identify factors associated with severity of buttock and leg pain

	Variable	*B*^a^	SE^b^	*β*^c^	*P*	Adjusted *R*^2^
Final	(Constant)	3.958	0.412			0.520
	NRS LBP	0.223	0.047	0.221	< 0.001	
	NRS Buttock and leg numbness	0.392	0.041	0.445	< 0.001	
	SF-36 Bodily pain	−0.037	0.006	−0.283	< 0.001	
	% Slip	0.028	0.011	0.100	0.015	

The independent variables entered into the model were age, sex, body mass index, presence and number of comorbidities, trunk muscle mass, appendicular skeletal muscle mass, NRS, SF-36 score, and radiological and MRI findings

^a^Unstandardized coefficient

^b^standard error

^c^standardized coefficients

**Table 5:** Stepwise linear regression analysis to identify factors associated with severity of buttock and leg numbness

	Variable	*B*^a^	SE^b^	*β*^c^	*P*	Adjusted *R*^2^
Final	(Constant)	−1.557	1.683			0.481
	Age	0.036	0.016	0.100	0.027	
	BMI	−0.181	0.037	− 0.221	< 0.001	
	Trunk muscle mass	0.158	0.036	0.212	< 0.001	
	NRS LBP	0.204	0.057	0.178	< 0.001	
	NRS Buttock and leg pain	0.595	0.056	0.524	< 0.001	
	Total number of Modic type 1 changes	0.635	0.289	0.096	0.029	
	Severity of stenosis (maximum stenotic level)	0.362	0.171	0.092	0.035	

The independent variables entered into the model were age, sex, body mass index, presence and number of comorbidities, trunk muscle mass, appendicular skeletal muscle mass, NRS, SF-36 score, and radiological and MRI findings

^a^Unstandardized coefficient

^b^standard error

^c^standardized coefficients

## Discussion

This study shows the strongest associations among LBP, buttock and leg pain and buttock and leg numbness, and the limited associations between LBP and physiological findings. However, trunk muscle mass, lumbopelvic alignment, and endplate defects, but not spinal stenosis are associated with the severity of LBP. The severity of spinal stenosis weakly correlated with the severity of buttock and leg pain and buttock and leg numbness and was associated with buttock and leg numbness in the regression analysis.

In this study, LBP and buttock pain were evaluated separately because LBP and buttock pain have different pain characteristics. A previous study reported that in patients with chronic lumbar spinal disorders, buttock pain was significantly associated with neuropathic pain regardless of the presence of leg pain, and LBP was associated with nociceptive pain rather than neuropathic pain [24]. Many studies have shown improvements in LBP after decompression surgery. One possible explanation for this is that LBP and buttock pain might be assessed together [25, 26].

In this study, buttock and leg pain and buttock and leg numbness, but not spinal stenosis evaluated according to the morphology of the dural sac were associated with the severity of LBP. Dural sac cross-sectional area in axially loaded MRI is significantly correlated with the severity of symptoms, which conventional MRI could not detect [27]. The use of conventional MRI might be insufficient to assess the severity of spinal stenosis, because symptoms are typically aggravated during walking or in the upright position in patients with LSS. Other possible explanation for this is that central pain-modulating mechanisms and pain cognitions have the important role in the associations among LBP, buttock and leg pain and buttock and leg numbness [4], because almost all patients in this study had the LSS symptoms for more than 3 months. Nociplastic pain and structural and functional changes in the brain can occur in individuals with chronic pain conditions that are primarily nociceptive or neuropathic [28]. It should be kept in mind that the pathoanatomical findings alone could not explain the LBP in patients with LSS.

On the other hand, the total number and severity of spinal stenosis were weakly correlated with buttock and leg numbness, and severity of spinal stenosis was associated with buttock and leg numbness in the regression analysis. These associations were not observed in pain such as LBP and buttock and leg pain. Chronic nerve root compression more than 6 months causes endoneurial fibrosis and Wallerian degeneration of nerve fibers [29]. Previous study showed that the improvement in leg numbness was less than those in leg pain after decompression surgery for LSS, and duration of symptoms and preoperative dural sac cross-sectional area were the predictive factors for residual leg numbness [30]. Therefore, buttock and leg numbness might be closely related to degeneration of nerve fibers caused by compression due to spinal stenosis.

Our study indicated that endplate defects, but not severity of spinal stenosis assessed on MRI images was independently associated with the severity of LBP. A previous population-based study showed that the presence of endplate defects was associated with lifetime back pain and with the intensity of the worst back pain, after adjusting for the effects of Modic endplate changes and disc degeneration [31]. Endplate degeneration has been reported to be an independent risk factor for disc degeneration and Modic endplate changes progression in a population with LBP [32]. In patients with LSS, the presence of endplate defects has also been found to be independently associated with the presence of LBP [12]. Our findings suggest that while vertebral endplate defects are an independent factor related to the severity of LBP, spinal stenosis itself does not contribute to LBP in patients with LSS. However, caution is needed when interpreting the results, because some of endplate defects were also shown in patients with no or mild LBP, as shown in the Fig. 1. Future studies should focus on whether preoperative endplate defects affect postoperative LBP after decompression surgery.

Trunk muscle mass and PT were also associated with severity of LBP in this study. Multifidus and erector spinae play important roles in maintaining lumbopelvic alignment such as PT, lumbar lordosis, and sacral slope [33]. Previous studies showed that fatty infiltration in the multifidus muscle caused by spinal stenosis was associated with functional status as measured by claudication distance and Oswestry disability index in patients with LSS [34, 35]. On the other hand, the severity or duration of back or leg pain have been reported not to be associated with multifidus morphology [35]. Although decreasing lumbar lordosis can relieve LSS symptoms, our study shows that there are no correlations between lumbopelvic alignment such as lumbar lordosis and PT, and leg pain and numbness. There is a possibility that LBP in patients with LSS is independently caused by atrophy of paraspinal muscle and lumbopelvic malalignment, without LSS symptoms or multifidus morphological change caused by LSS. Future studies are needed to determine whether multifidus morphological change is caused by LSS itself or whether paraspinal muscle atrophy results from aging or physical inactivity due to neurogenic claudication associated with LBP in patients with LSS. Moreover, clinical trials that assess the effectiveness of exercise therapy focused on paraspinal muscle in patients with LSS are expected.

Our study showed that multifactorial factors, not radiological stenosis affected LBP in patients with LSS. To improve clinical outcomes in patients with LSS, LBP should be carefully assessed, including degenerative spine conditions, spinopelvic alignment, trunk muscle, and pain perception. Furthermore, investigating the impact of surgery and/or exercise therapy on those factors and LBP can help better understand LBP in patients with LSS.

This study has some limitations. First, the cross-sectional design limits causal inference. Second, the timing of MRI, in addition to the need for in-supine positioning without weight bearing, is another limitation. LSS symptoms are often secondary to inflammatory flareups that occur affecting any of the lumbar structures, such as intervertebral disc herniation, capsular and ligament hypertrophy, and synovial cysts. Psychological factors such as depression, anxiety, pain catastrophizing, and fear avoidance beliefs were not assessed. Those assessments might help with interpreting our findings, because patient reported outcomes had stronger correlations with pain and/or numbness than imaging findings. Patients with severe kyphosis that might be affect the results were not excluded. In this study, physical functioning assessments were not measured. Previous study found that trunk muscle mass measured BIA is strongly correlated with back muscle strength in elderly population [36]. Assessment of back muscle strength might help better understand LBP in patients with LSS. Finally, while we showed that appendicular and trunk muscle mass and radiological findings such as PT, PI-LL, and total number of endplate defects were significantly correlated with the severity of LBP, Spearman’s rank correlation coefficients were low. LBP is a multifactorial problem associated with several biophysical factors, including inflammatory cytokines, altered pain-processing mechanisms and central sensitization, and psychological, social, and genetic factors [4, 37]. Nevertheless, despite our data showing high variability (multivariate regression analysis R^2^ = 0.370), our relatively large sample size (*n* = 293) showed that trunk muscle mass, PT, and endplate defects, but not spinal stenosis were associated with the severity of LBP in patients with LSS.

## Conclusions

The strongest correlations were observed among LBP, buttock and leg pain and buttock and leg numbness, and there were only limited correlations between LBP and physiological findings. Age, female sex, trunk muscle mass, diabetes, buttock and leg pain, buttock and leg numbness, SF-36 vitality, PT, and total number of endplate defects were associated with the severity of LBP. The severity of spinal stenosis was associated with the severity of buttock and leg pain or numbness, but not with the severity of LBP. Collectively, trunk muscle mass, lumbopelvic alignment, and endplate defects, but not severity of stenosis are partly associated with the severity of LBP, but buttock and leg pain and buttock and leg numbness have strongest relationships with LBP in patients with LSS.

## Data Availability

The datasets used and/or analysed during the current study are available from the corresponding author on reasonable request.
